# Rudimentary Preparation, Compared with Higher Education, for Specialties in Medicine and Surgery*President’s address, American Academy of Medicine, Baltimore, May 4, 1895.

**Published:** 1895-07

**Authors:** J. McFadden Gaston

**Affiliations:** Atlanta, Ga.


### RUDIMENTARY PREPARATION, COMPARED WITH HIGHER EDUCATION, FOR SPECIALTIES IN MEDICINE AND SURGERY.*

BY J. McFADDEN GASTON, M.D.,

ATLANTA, GA.

(*Concluded from June number.*)

The therapeutic application of drugs depends upon a knowledge of the normal and abnormal conditions of the animal functions. While the term physiology is applicable to all organized substances and hence general in its operation, biology expresses more definitely the vital phenomena presented in the normal state of living matter, and must be comprehended by the student before he can understand the departures from the standard of health known as disease. In other words, the transition from the physiological action to the pathological disorder of the system must become the basis for the use of remedies; and affords the only scientific explanation of therapeusis. A mere empirical treatment of disease has no rational foundation, but rests entirely upon the observation

*President's address, American Academy of Medicine, Baltimore, May 4, 1895.

that certain classes of drugs, when given in definite forms of disease, produce salutary results; and hence are indicated in similar cases. This proceeds upon the supposition that like causes produce like results, without a recognition of many modifying elements.

There are very few, if any, specifics for diseases, and the student should be taught to interpret the indications for special means of relief, without any regard to a stereotyped rule of treatment in given forms of diseases. The therapeutic instruction should be founded upon those general principles, which imply a knowledge of physiology, pathology, and materia medica, including pharmacy. The professor who limits therapeutics to the routine use of private formulas in certain diseases, falls far short of the high standard of teaching, which the lights before us at the present day should lead us to expect. All are aware that therapeusis is not confined to the employment of drugs, and in many instances the result depends quite as much upon the diet and regimen as upon the medicine. But touching the medicinal treatment of any disorder, I protest strenuously against that mode of instruction which tends to empiricism rather than to secure a rational foundation for therapeutics.

There is a prevalent impression among the members of the medical profession, that a very distinct line is to be drawn between the practice of medicine and surgery, by the relative demand for medication in the former as compared with the latter. Indeed, the adoption in European schools of the terms internal and external, for distinguishing the practice of medicine from that of surgery, implied a recognition of this division ; and even in this country, the use of drugs has been relegated very generally to the physician.

For a proper view of the respective fields of service occupied by the physician and the surgeon, I have elsewhere defined the duties of the former as limited to the correction of the functional derangements of the system; while the sphere of the latter is confined to organic or structural changes in any portion of the body. Any measure, whether operative or otherwise, which may conduce to the relief of these modifications of structure, must come, therefore, within the domain of surgery.

Local congestion and engorgement of tissues are not considered as structural changes; but whenever depositions occur, which materially alter the size and density of the part involved, it becomes an organic modification and falls under the category of surgical pathology.

This altered condition may be amenable to medication, and yet none the less surgical; so that the use of internal remedies is often requisite in treating a surgical case, in advance of an operation or subsequently, in the treatment of the patient.

The course of instruction in surgery should emphasize the fact, that surgical disorders may occur in the parts of the organism which lie internally or externally, and that they may be treated by internal or external means; and that they are not limited to mechanical appliances.

The student should have it impressed on his mind, that all the complications growing out of an organic modification of tissues belong to the department of surgery, and must be taken into account in the treatment of the case.

The student should not learn simply how to cut, but also where to cut, and when to cut, as well as when to withhold the knife.

Surgical pathology ought to occupy a very conspicuous place in the preparation of the student for surgical work. In like manner surgical diagnosis presents an urgent claim to his consideration; and in these latter days when the cranium, the thorax, and the abdomen are becoming the workshops of the surgeon, it behooves the teacher to lay great stress upon the recognition of the disorders of their respective contents. A thorough knowledge of the part involved, including its relations to adjoining structures in the normal state, is essential for detecting any departure from this standard in disease; and no student who is well grounded in anatomy and physiology ought to be at a loss in discerning the difference between the normal and abnormal structures. The microscope and other means, with certain instrumental procedures, are frequently requisite for a correct surgical diagnosis; and should enter into the rudimentary preparation for higher work.

If the dictum of a famous gynecologist should be generally accepted, in case of doubt as to the nature of any trouble within the abdomen of a woman, we would cut in and find out the origin of the trouble; and then make a radical operation or not, as might seem proper. I have witnessed such an exploratory procedure by the most distinguished operator in this country, and, nothing being found, the woman was left in blissful ignorance of the result, and claimed afterwards to have been greatly relieved of her pelvic trouble. While an exploratory operation may be sometimes warranted, and becomes emphatically a typical surgical diagnosis, we may lay it down as a rule, that masterly inactivity is preferable to laying open the skull, the chest, or the belly, for the purpose of discovering the nature of the internal disorder. I had a most untoward experience in former years from the use of the trephine for the removal of bone in a perforated fracture of long standing, which was thought to be the cause of epilepsy, when no projection was found, and my patient died from this simple operation.

An abundance of caution should be inculcated upon students in regard to surgical diagnosis of this character, and while circumstances warrant the resort to exploratory operations by skillful surgeons in some cases, it would be nothing less than criminal rashness in those having a limited experience in surgery. There are ways and means, however, independent of such procedures, to ascertain, with a reasonable degree of certainty, the pathological conditions involved in a given case; and in the course of instruction the most minute details should be dwelt upon, to elucidate such points as may effect an assured diagnosis before undertaking any surgical operation of magnitude. In view of the errors committed by distinguished surgeons from lack of proper precautions in making an examination of the case in hand, it is held that surgical diagnosis should occupy a more prominent place in our medical colleges than has been awarded to it heretofore. I know of what I speak, and if all the revelations of the errors in diagnosis made by surgeons were brought to light, they would suffice to impress this point.

The bearing or relative influence of the different departments of medical instruction upon each other is of such a nature, that all points should be impressed upon the mind of the student at the different stages of his course of lectures. To accomplish this satisfactorily undue prominence should not be given to any special branch; but all should receive due attention, by the grading of the subjects of study in a three or four years' course of lectures. With this rule laid down to guide the medical student, he should not entertain the thought of devoting himself to a specialty until after graduating in the regular curriculum of the medical college. He should have received, during his three or four years' application, a fair knowledge of all that is taught, and be prepared to enter upon the duties of a general practitioner in medicine and surgery; thus being prepared to determine the special line of service to which his attention may be given subsequently, and also having the proper qualification for entering upon the study of matters which shall fit him for it. It is not desirable that a student, at the outset of his medical course, shall determine to be a specialist; as this must warp his mind, in carrying on the different branches of the curriculum, towards those things which may promote his specialty, and lead to the neglect of other matters of paramount importance. After all the usual branches are fully mastered, and the student is in a position to exercise a choice of that field of life-work which is best suited to his tastes and capacity, he may be fitted for selecting a specialty in medicine or in surgery. Then and not before, provision may be made for taking up that line of study which may be requisite to qualify him for the special work which is before him. The specialist should not look upon his field of service as requiring less preparation than is demanded for the general practitioner of medicine and surgery; but as exacting a higher order of qualification by the addition of those attainments which are conducive to the proper discharge of the special service in his line.

This is an age of specialists, and we have in active operation fourteen distinct national organizations for the development of their respective views; while outside of these societies, other associations, for co-operation in different special fields, have been formed by those who are interested in circumscribing their labors. It is not necessary to enumerate the titles of these several bodies of specialists, as the fellows of the academy are presumed to be familiar with them; but it may be stated that they cover almost every phase of investigation which is comprehended by medical science in its widest and most extended acceptation. Even our great American

Medical Association, which includes all classes and conditions of medical men, is, for all practical purposes, divided into sections of specialists, which operate independently of each other; and thus keep up the lines of separation between those members of the profession who are occupied in different departments of practice, having very limited professional relations with other practitioners.

The work of preparing medical men for specialists is met to a greater or less extent by the organization of polyclinics and post-graduate schools; and yet the limited attendance of the patrons of these institutions upon their brief courses of instruction impairs materially their utility. Unfortunately for all concerned, the rushing spirit crops out in everything at the present time, even from a boy pressing his suit with his sweetheart to the disciple of Esculapius who makes haste to secure his profession. If our people could adopt the German idea of thoroughness in executing what is undertaken, with the observance of the motto *festina lente*, it would redound to the building up of a solid foundation for the profession, by completeness and exactness in medical education. The graduate of a college who enters upon the study of some specialty in a polyclinic or post-graduate school should avail himself of all that can be learned in the special clinical course, and attend hospital service, illustrating the special work in view.

The post-graduate school should embody the highest order of instruction in all the different departments of medicine and surgery, as the teacher is supposed to be competent to direct the minds of those already well versed in medical knowledge to greater achievements in the special branch of their selection. The very best talent of the profession ought, therefore, to be employed in the work; and as a matter of fact, some of the professorships are filled by first-class men. But it must be admitted that many of the places are occupied by inferior men, who reflect no credit upon the undertaking, and have not conduced to the elevation of the medical profession. This, however, is no objection to the principle upon which they are based, and as few will attend the service of those who are inefficient, this must be a corrective in the working of these schools. Independent of the patronage by specialists, a well regulated polyclinic should attract those members of the profession who desire to keep abreast of the advances in the different departments of medical science, and their success is a matter for congratulation. The fad, which has taken hold of a large number of medical graduates to visit schools abroad, where the language is unknown, and look upon the work that is done, without comprehending the pantomime enacted before them, seems to be at least a waste of time and a useless expenditure of money. If home institutions do not fill the ambition of these aspirants to fame, and they must seek a foreign field, it is far better for them to go to London or Edinburgh than to Paris, Vienna, or Berlin to complete their special studies. Any one who goes to the latter, without being acquainted with the language of the country, will require months, if not years, to become sufficiently familiar with it to profit by the lectures of the teachers, and few stay long enough to master the foreign tongue.

It can, therefore, avail little to make a stay of less than two years for the study of a specialty in a foreign school; and this sojourn is more for the *éclat* of kneeling at the feet of a teacher of great reputation than for any substantial advantages gained from his instructions, while greater prestige would attend a more diligent use of the opportunities afforded here in our polyclinics.

In the evolution of specialism it may be anticipated that little or nothing will be left for the occupation of the general practitioner; and it becomes a grave question as to the final outgrowth of this division of labor to such an extreme degree. Many of us, after long years of practice have not seen our way clear to entering upon a specialty, and I am fully convinced that the rôle of the general practitioner is essential for the advancement of medicine and surgery. It will prove disastrous to the best interests of the medical profession, that all practical work shall be parceled out into these subdivisions; and hence, I would insist that the curriculum of instruction in our medical schools should be maintained, so as to qualify their graduates for entering upon general practice in medicine and surgery. There should not be any provision in the graded courses of three or four years, for turning out specialists; but the preparation for a specialty should begin after receiving the title of

M.D., which is only intended to indicate fitness for general practice.

It is a matter for serious consideration whether any considerable number of the medical colleges in the United States are in a condition to enter upon a four years' graded course at the present time or within the near future. While the endowed institutions are prepared to assume the responsibilities of such a step, and it is commendable in independent organizations to afford the very best opportunities for the highest attainments of those who can devote this additional time to preparatory training for the medical profession, a large majority of the medical colleges cannot undertake more than a three years' graded course. They are ready to make a decided advance by the adoption of the three years' graded course; and many of them have already entered upon the curriculum of instruction on this basis. This is so marked an improvement upon the old two years' course that we should congratulate the profession upon this evidence of progress, and be content to await further developments to authorize an increase of another year. We should be satisfied to let well enough alone and not run the risk of disaster by such a great change so suddenly.

In consideration of the various points presented, it may be concluded that aside from a preliminary literary qualification, the study of medicine should embrace at least one year of instruction under a preceptor, three or four years of graded courses of six months each in a medical college as a requisite for the degree of M.D.; and for those intending to practice a specialty, a minimum of one year's attendance upon a special course in a post-graduate school. Anything short of this program of studies for the medical profession, must leave the student without proper equipment for his work, and bring to him only chagrin and disappointment in entering upon the responsibilities of practice. If my ideal of thorough preparation for the duties of the physician and surgeon can be fully realized, the practitioner ought to be competent to solve the problems of pathology, diagnosis, and therapeutics, upon taking charge of a case, and not expect to learn how to treat disease by his unfortunate experience at the bedside of his suffering patient.

